# Availability, Prices and Affordability of Antibiotics Stocked by Informal Providers in Rural India: A Cross-Sectional Survey

**DOI:** 10.3390/antibiotics11040523

**Published:** 2022-04-14

**Authors:** Meenakshi Gautham, Rosalind Miller, Sonia Rego, Catherine Goodman

**Affiliations:** Department of Global Health and Development, Faculty of Public Health and Policy, London School of Hygiene and Tropical Medicine, 15–17 Tavistock Place, London WC1H 9SH, UK; rosalind.miller@lshtm.ac.uk (R.M.); sonia1rego@yahoo.com (S.R.); catherine.goodman@lshtm.ac.uk (C.G.)

**Keywords:** antibiotics, affordability, medicine prices, markups, informal providers, private sector, rural India, access, watch, AWaRe

## Abstract

Providers without formal training deliver healthcare and antibiotics across rural India, but little is known about the antibiotics that they stock. We conducted a cross-sectional survey of such informal providers (IPs) in two districts of West Bengal, and assessed the availability of the antibiotics, as well as their sales volumes, retail prices, percentage markups for IPs and affordability. Of the 196 IPs that stocked antibiotics, 85% stocked tablets, 74% stocked syrups/suspensions/drops and 18% stocked injections. Across all the IPs, 42 antibiotic active ingredients were stocked, which comprised 278 branded generics from 74 manufacturers. The top five active ingredients that were stocked were amoxicillin potassium clavulanate (52% of the IPs), cefixime (39%), amoxicillin (33%), azithromycin (25%) and ciprofloxacin (21%). By the WHO’s AWaRe classification, 71% of the IPs stocked an ACCESS antibiotic and 84% stocked a WATCH antibiotic. The median prices were in line with the government ceiling prices, but with substantial variation between the lowest and highest priced brands. The most affordable among the top five tablets were ciprofloxacin, azithromycin, cefixime and amoxicillin (US$ 0.8, 0.9, 1.9 and 1.9 per course), and the most affordable among the syrups/suspensions/drops were azithromycin and ofloxacin (US$ 1.7 and 4.5 per course, respectively), which are mostly WATCH antibiotics. IPs are a key source of healthcare and antibiotics in rural communities; practical interventions that target IPs need to balance restricting WATCH antibiotics and expanding the basket of affordable ACCESS antibiotics.

## 1. Introduction

The recognition of antimicrobial resistance (AMR) as a public health emergency has drawn attention to the need for the sustainable use of antibiotics [1]. Globally, increasing rates of AMR predict a frightening scenario, where treating common infectious diseases will become difficult, costly and prolonged, and multidrug resistant infections will increase, causing an estimated 10 million deaths annually by 2050 [2]. In 2019, 1.27 million deaths were directly attributable to AMR, with the highest burdens in sub-Saharan Africa and South Asia [3]. India, where this study is located, is among the highest AMR-prevalant countries [4], with 50% or more of *E. coli*, and 30% or more of *Klebsiella* in rural and urban settings, which are resistant to ciprofloxacin (a quinolone), and first-, second- and third-generation cephalosporins [5,6,7], which are the first-line antibiotic treatments for bacterial diarrhoea and typhoid fever.

The excessive and inappropriate use of antibiotics in humans and animals is an important driver of AMR [8]. However, it is also recognised that people in many parts of the world do not have access to essential antibiotics, and that there may be more deaths due to a lack of antibiotic access than due to drug-resistant infections [9]. To encourage appropriate antibiotic use and equitable access, the World Health Organization’s essential medicine list provides a three-tiered risk-based antibiotic classification (ACCESS, WATCH and RESERVE), with recommendations about availability and use that are specific to each tier [10]. 

ACCESS antibiotics should be available widely as first- and second-choice antibiotics for the management of common clinical syndromes; WATCH antibiotics should be used sparingly and should be carefully monitored; and RESERVE antibiotics should be used as a last resort to treat drug-resistant infections when all other antibiotics have failed.

Furthermore, to reduce antibiotic misuse, the WHO’s Global Action Plan for Antimicrobial Resistance [1] recommends that, ‘the distribution, prescription, and dispensing of antimicrobials is carried out by accredited health or veterinary professionals under statutory body supervision or other suitably trained person authorized in accordance with national legislation.’ However, the nonprescription sale of antibiotics is common [11], and it accounts for 19 to 100% of the antibiotic use across countries [12], with some of the highest levels (50–100%) reported from Asian countries, including Vietnam, Bangladesh and India [13,14,15]. Nonprescription antibiotic sales take place in both licensed pharmacies [16,17] and unlicensed outlets. For example, in a mapping of the antibiotic suppliers in six low- and middle-income countries (LMICs) in Asia and Africa, 65% of the providers in Vietnam, and 52% in Bangladesh, did not have legal authorisation [15]. In many LMICs, private vendors, including informal ones, are better stocked than many government health facilities [18]. In the Cameroon and the Democratic Republic of the Congo, for example, 98% of the retail pharmacies and informal vendors stocked selected ACCESS and WATCH antibiotics, compared to only 62% of the government facilities [19].

For the large numbers of people in LMICs who lack access to the formal health system, informally trained private healthcare providers are an important source of primary care at first contact, especially for mild illnesses [15]. Even pharmacies can be limited in rural areas and they can be located only near main roads, far from the more remote villages [17]. Informally trained healthcare providers provide drugs as well as case management in many such areas. They include rural medical practitioners and village doctors in India and Bangladesh [20,21], and unregistered pharmacies, drug shops and itinerant health providers and drug sellers in Indonesia, Cambodia, Uganda and Nigeria [22,23,24,25,26]. In India, an estimated 56% of all healthcare providers lack formal medical qualifications [27]. In some regions, these informal providers (IPs) account for 70% of the health provider visits by rural households [28]. In rural central India, for example, 73% of healthcare seeking for sick under-5 children (in a community cohort, followed over a one-year period) was from local IPs [29]. IPs function out of small clinics and shops, or they are itinerant and mobile, charging a fee for services, which include the antibiotics and other drugs that they dispense, and typically without an authorized prescriber’s prescription [30,31]. IPs may lack formal recognition, but they are targeted by pharmaceutical representatives for drug promotion, and by private doctors for training and mentorship in exchange for patient referrals [31,32,33,34]. However, there are shortfalls in their antibiotic knowledge and practice [34,35,36], and their antibiotic-dispensing practices have proven difficult to change through training alone [35].

Data on the availability and use of antibiotics from nonformal sources in India, or in other countries, remain scarce. The standardised data collection approaches for antibiotic availability and use are limited to registered retail outlets and health facilities [18,37,38] and fail to include the more informal outlets, which are more challenging to access and survey. These missing data on the informal sector are increasingly acknowledged as being critical to a thorough understanding of the antibiotic consumption. The WHO’s report on the global surveillance of antibiotic consumption states that, ‘no data explicitly capturing antimicrobials circulating on the informal market have been obtained’, and acknowledges that ‘for countries in which the informal market is significant, only an incomplete picture of antibiotic consumption can be presented’ [39]:

In India, the role of IPs as a key source of antibiotics is well documented, but only one study has quantified the antibiotics that are dispensed/prescribed by a small number of IPs (12 IPs over 18 months) [40]. Moreover, despite the key role of financial incentives in influencing IP and user practices [31], no data are available on IPs’ antibiotic prices, the mark-ups or the affordability to users. We conducted a cross-sectional survey to address this evidence gap, adapting the WHO/HAI methodology [37] to assess the availability and sales of the antibiotics that were stocked in IP clinics, their retail prices, their mark-ups for IPs and their affordability compared to the daily wage of the lowest paid unskilled worker. This antibiotic audit was part of a larger study on the drivers of antibiotic use by the rural IPs in the districts, South24Parganas and Birbhum, in the state of West Bengal in India [31].

## 2. Results

We identified 291 IPs across our two study sites: 147 in Birbhum and 144 in South24Parganas. Of the 291 IPs, 16 said that they only prescribed and did not dispense antibiotics; a further 42 usually dispensed but did not have any antibiotics in stock on the day of the visit; and 37 were unwilling to disclose their stock. We therefore collected antibiotic stock data from 196 IPs: 104 in Birbhum and 92 in South24Parganas. Nearly all the IPs were male (98%) and 54% were between the ages of 35 to 55 years (Table 1). A total of 65% had completed schooling up to Class 10 or 12, and 35% had completed schooling plus graduation or postgraduation. A total of 72% had allied health or paramedical certification, mostly in basic primary healthcare (62%), pharmacy science (9%) or as laboratory assistants (1%) (Supplementary Table S1). These courses were offered by local nongovernmental organisations and they did not fall under the ambit of the ‘legal’ qualifications that are required to practice medicine in India. A total of 54 IPs (28%) had no such certification, and around 40% of these practiced in two of the most remote and difficult-to-access blocks of South24Parganas. A total of 83% had learned their practical medical skills by working as assistants to formally trained doctors. They had their own well-established independent practices; 68% had been in practice for 10 years or more. All of the IPs operated out of solo clinics that were typically open 6–7 days a week, with most of the IPs living and working in the same premises or nearby. They operated as neighbourhood general practitioners who provided outpatient care to local patients for a range of communicable and noncommunicable conditions. A total of 40% said that they treated rural backyard animals too, such as small poultry and cattle. All of them used antibiotics in their treatment practices; 95% said they directly dispensed antibiotics, while 86% said they also prescribed.

### 2.1. Antibiotic Availability and Sales Volumes

Among the 196 IPs, 84% had antibiotic tablets in stock, 74% had syrups/suspensions/drops and 18% had injections. The IPs stocked a median of two antibiotic active ingredients in tablet form (IQR: 1 to 3), and one syrup/suspension/drops (IQR: 0 to 2) (Supplementary Table S2). As relatively few IPs stocked injections, the median number of active injection ingredients that were stocked was 0.

**Figure 1:**
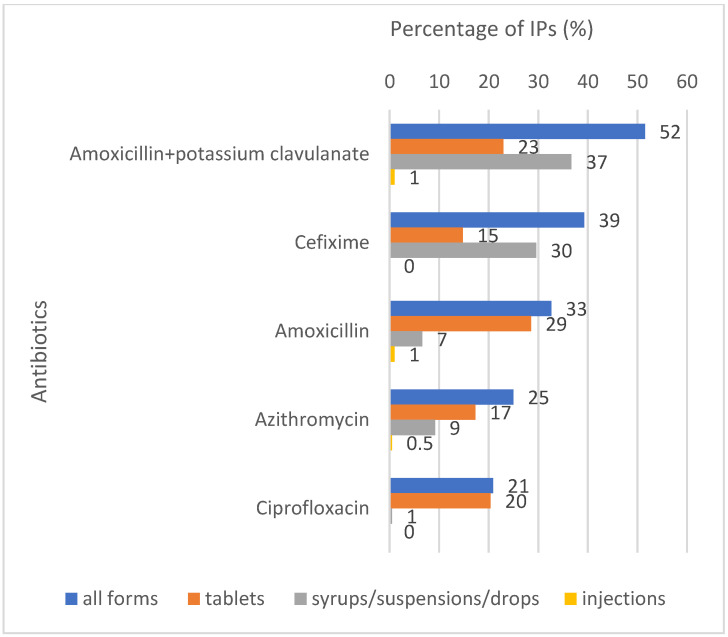
Top 5 antibiotic active ingredients (all formulations) stocked by IPs (*n* = 196).

Across all IPs, we documented 42 antibiotic active ingredients, across all formulations. The top five are shown in Figure 1 (for a complete list, see Supplementary Figure S1). The five most common active ingredients (across all formulations) were amoxicillin potassium clavulanate (stocked in 52% of IP clinics), cefixime (39%), amoxicillin (33%), azithromycin (25%) and ciprofloxacin (21%). However, the top five varied by formulation, with amoxicillin being the most common for tablets, amoxicillin potassium clavulanate being the most common for syrups/suspensions/drops and amikacin being the most common for injections (Table 2).

**Figure 2:**
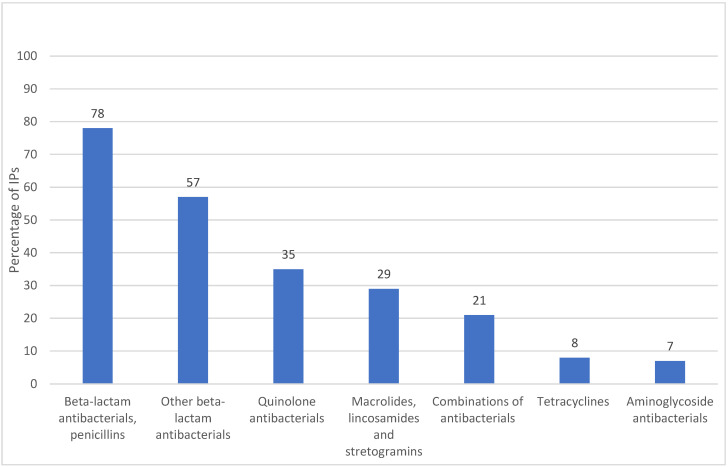
Percentages of IPs stocking antibiotics by the WHO anatomical therapeutic chemical (ATC) classification system [41] (*n* = 196).

We grouped all of the available antibiotics by their anatomical therapeutic chemical (ATC) classification, which is a system that classifies drugs by their pharmacological and therapeutic properties [41] (Figure 2). The most commonly stocked ATC class was ‘beta-lactam antibacterials, penicillins’ (78% of IPs), which include amoxicillin, ampicillin and their combinations, followed by ‘other beta-lactam antibacterials’ (57%), which include cephalosporins, such as cefalexin, cefuroxime and cefixime. ‘Quinolones’ were stocked by 35% of the IPs, ‘macrolides, lincosamides and stretogramins’ by 29%, ‘tetracyclines’ by 8% and ‘aminoglycoside antibacterials’ by 7%. A total of 21% of the IPs stocked antibiotic combinations.

**Figure 3:**
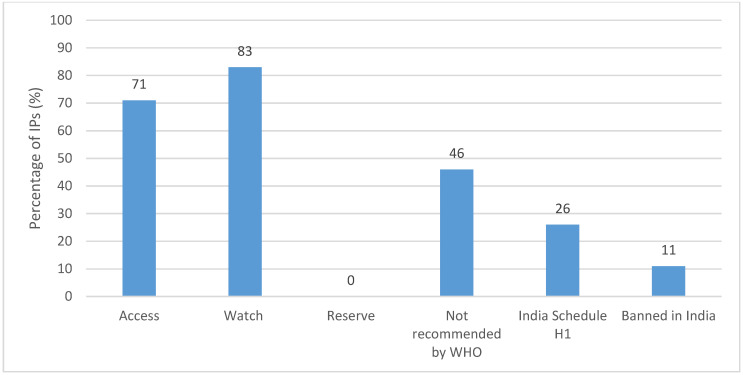
Percentages of IPs stocking antibiotics by WHO AWaRe classification and Indian regulatory * status (*n* = 196).

We further compared all of the available antibiotics with the WHO’s AWaRe list [10]; with the list of combinations not recommended by WHO [10]; with Schedule H1 of the Indian Drugs and Cosmetics Act, which includes 46 restricted drugs, including several third- and fourth-generation antibiotics, and is more strictly monitored than the other prescription drugs in Schedule H [42]; and with a list of banned drugs in India [43] (Figure 3) (for product details, see Supplementary Table S3). We found that 71% of the IPs stocked an ACCESS antibiotic, and 83% stocked a WATCH antibiotic, but none stocked a RESERVE-category antibiotic. Combinations that are not recommended by the WHO were stocked by 46% of the IPs: a total of 26% stocked a Schedule H1 antibiotic, and 11% stocked an antibiotic that is banned in India. The list of drugs/antibiotics banned in India was different from the list of antibiotic combinations not recommended by the WHO; the former included only three from the WHO list (details shown in Supplementary Table S3). The H1 list of antibiotics was also different from the WATCH list in that only 5 of the 16 WATCH antibiotics that were stocked in the IP clinics were included in Schedule H1.

Beta lactam antibacterials, penicillins (J01C): examples include amoxicillin, ampicillin and amoxicillin + potassium clavulanate.

Other beta-lactam antibiotics (J01D): examples include cefixime, cefpodoxime and cefadroxil.

Quinolone antibacterials (JO1M): examples include moxifloxacin, norfloxacin, ciprofloxacin, ofloxacin and levofloxacin.

Macrolides, lincosamides and stretogramins (J01F): examples include roxithromycin, erythromycin, lincomycin and azithromycin.

Combinations of antibacterials (J01R): examples include azithromycin + cefixime, ofloxacin + ornidazole, cefixime + ofloxacin and ciprofloxacin + tinidazole. However, only two of these combinations—ciprofloxacin + tinidazole and norfloxacin + metronidazole—are listed under the J01R combinations. The rest are irrational combinations.

Tetracyclines (J01A): examples include doxycycline, oxytetracycline and tetracycline.

Aminoglycoside antibacterials (J01G): examples include amikacin and gentamycin.

Note * Including antibiotics in Schedule H1 of the Indian Drugs and Cosmetics Act. This Schedule contains a restricted set of 46 prescription drugs, which include third- and fourth-generation cephalosporins, carbapenems, newer fluoroquinolones and first- and second-line antitubercular drugs. Sales of Schedule H1 drugs are more strictly monitored than those of Schedule H drugs [42]. *Banned drugs include drugs that are prohibited for manufacture and sale through gazette notifications under Section 26a of the Drugs and Cosmetics Act (1940) by the Ministry of Health and Family Welfare* [43].

For each of the top five antibiotics for the different formulations (Table 2), we present the median defined daily doses (DDDs) that were in stock on the day of the visit, the median reported DDDs that had been dispensed in the last seven days (by those who dispensed that product) and the median reported number of patients who had been dispensed the antibiotic in the last seven days (Table 3). Even though these were the top five antibiotics stocked, the volumes of stock and the sales per IP were low. For tablets, the median DDDs stocked per product in stock ranged from 10 to 25 DDDs, while the median that had been dispensed over the last seven days per product in stock ranged from 7 to 20 DDDs. Among the syrups/suspensions/drops and injections, the numbers were lower. For syrups/suspensions/drops, the median DDDs stocked per product ranged from 1.5 to 4 DDDs, and the median dispensed over the last seven days ranged from 2 to 6 DDDs. Compared to the tablets and the syrups/suspensions/drops, few IPs stocked injections: the median DDDs stocked ranged from 0.19 to 2.29, and the median DDDs dispensed ranged from 0.31 to 2.94. The median number of patients that had received a given product in the last seven days was 4 to 7 for tablets, 2 to 4 for syrups/suspensions/drops and 3 to 17 for injections. Among both tablets and syrups/suspensions/drops, the greatest number of DDDs that were stocked and that had been dispensed in the last seven days was for azithromycin, and, among the injections, it was for ampicillin.

### 2.2. Brands, Prices and Markups

We documented a total of 278 brands stocked (across all 42 antibiotics): 118 for tablets, 141 for syrups/suspensions/drops and 19 for injections, which were produced by 74 different manufacturers from both Indian and global pharmaceutical companies. These were brands of off-patent generic products. Table 4 provides the brand details for the top five antibiotics that were stocked for tablets and syrups/suspensions/drops. For tablets, the number of brands ranged from 22 for azithromycin to 11 for amoxicillin, and for syrups/suspensions/drops, the number ranged from 28 for amoxicillin potassium clavulanate to 7 for azithromycin. There were far fewer brands for injections, which ranged from just 1 to 3 for each of the top five stocked (data not shown because of small number of products). While there were many different brands available for each antibiotic, only 12% of the IPs stocked more than one brand of a given active ingredient formulation (6% for syrups and 6% for tablets).

**Figure 4:**
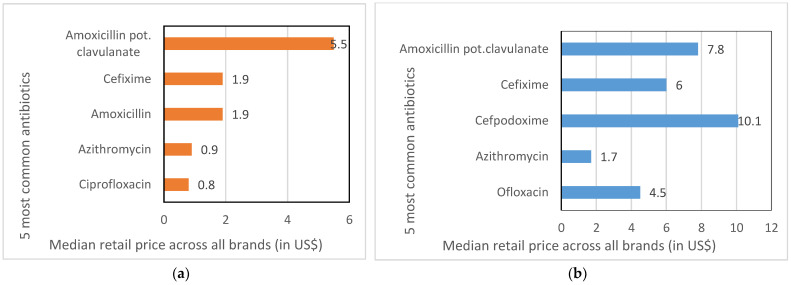
Median retail prices for standard courses of treatment across all available brands for the top five antibiotics stocked, by formulation. (**a**) Tablets (**b**) Syrups/suspensions/drops.

The median retail prices for standard courses of treatment for an adult for the five most common antibiotics, for tablets and for syrups/suspensions/drops, are presented in Figure 4. Amoxicillin potassium clavulanate, an ACCESS antibiotic, was the most expensive, in both tablet formulation (US$ 5.5) and as syrups/suspensions/drops (US$ 7.8). The cheapest were azithromycin (tablets: US$ 0.9; syrups/suspensions/drops: US$ 1.7) and ciprofloxacin tablets (US$ 0.8). For injections, we calculated prices per DDD (and not for a standard course of treatment), as the standard therapeutic durations were not clearly available for injections. The price for injections was higher per DDD for cefotaxime (USD 4.18), amikacin (US$ 2.39) and ceftriaxone (US$ 2.34), and it was lower for ampicillin (US$ 1.57) and gentamicin (US$ 1.11).

There was substantial variation in the median price across the lowest and highest priced brands, particularly for some of the top five antibiotics, for both tablets and syrups/suspensions/drops (Table 4). Among tablets, the ratio of the highest-to-lowest priced brand was greatest for amoxicillin potassium clavulanate at 6.2, followed by ciprofloxacin at 5.6, with the lowest ratio for amoxicillin at 2.2. For syrups/suspensions/drops, the ratio of the highest-to-lowest priced brand was greatest for cefpodoxime at 6.3 and for ofloxacin at 4.9, while the lowest was 1.7 for amoxicillin potassium clavulanate.

**Figure 5:**
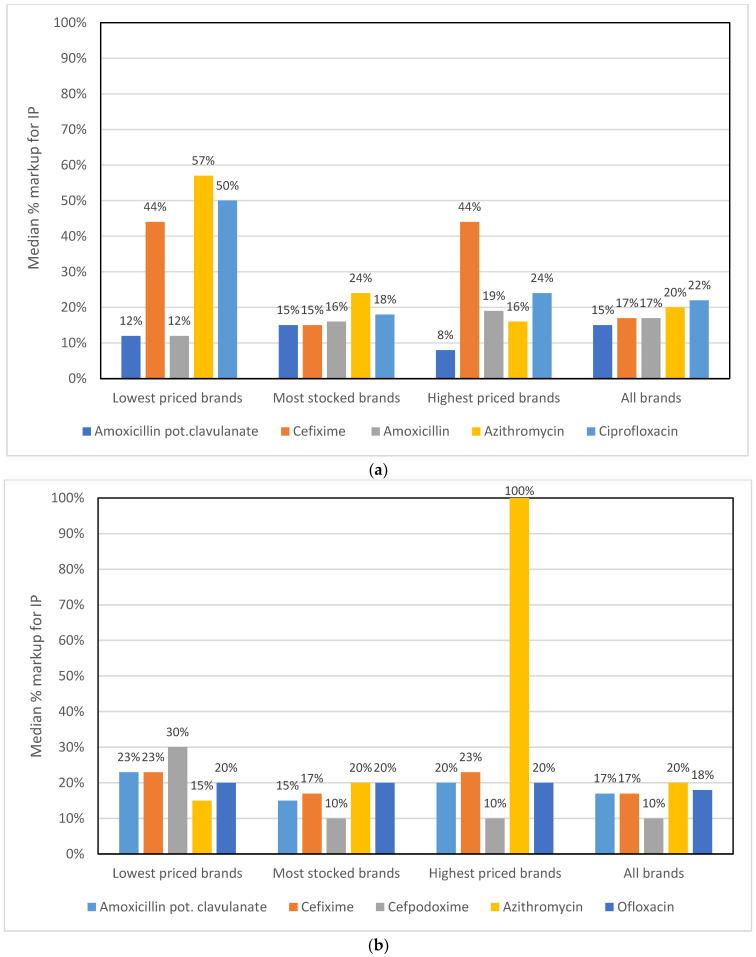
Median percentage markups for IPs for the top 5 antibiotics. (**a**) Tablets (**b**) Syrups/suspensions/drops.

The median percentage mark-ups ((sales price—purchase price)/sales price)) × 100) for the IPs for the top five antibiotics ranged from 15 to 22% for tablets, and from 10 to 20% for syrups, when all brands were included (Figure 5). However, there was considerable variation in the mark-ups between the lowest and highest priced brands for a given antibiotic, especially for tablets: from 12% to 57% for the lowest priced brands, and from 8% to 44% for the highest priced brands, but a more uniform 15% to 24% on the most stocked brands. The mark-ups for the different syrup/suspension/drop brands were, overall, more consistent than those for tablets, except for the highest priced brand (azithromycin), which had an outlier with a 100% mark-up.

### 2.3. Affordability

**Figure 6:**
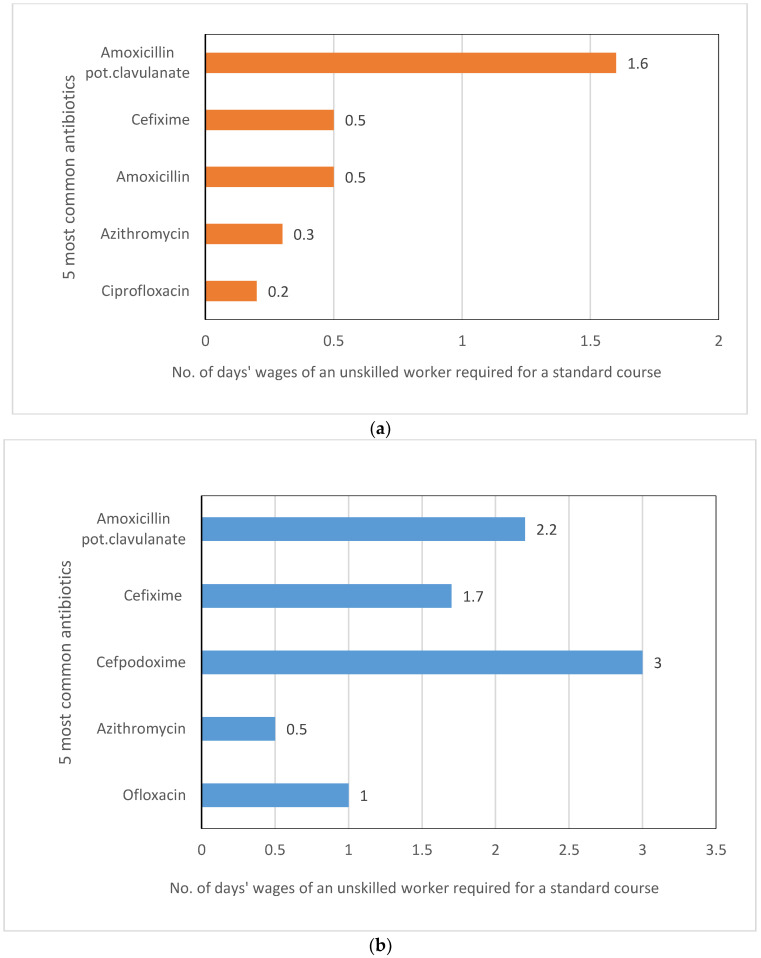
Number of days’ wages of an unskilled worker required to purchase a standard course of treatment for the five most commonly stocked antibiotics (across all brands), by formulation. (**a**) Tablets (**b**) Syrups/suspensions/drops.

We estimated the local affordability of each of the top five antibiotics (tablets and syrups/suspensions/drops) by calculating the number of days’ wages of an unskilled agricultural worker in West Bengal in 2017 [45] that would be required to cover the median price for a standard course of treatment for an adult across all brands (Figure 6). According to the WHO/HAI cutoff [37], a treatment course is unaffordable if it requires more than a day’s minimum wage. Of the tablets, only amoxicillin potassium clavulanate would be considered unaffordable (1.6 times a day’s wage). Three of the top five syrups/suspensions/drops would be considered unaffordable, with cefpodoxime requiring 3 days’ wages, followed by amoxicillin potassium clavulanate (2.2 days) and cefixime (1.7 days). The most affordable among the tablets were ciprofloxacin, azithromycin, cefixime and amoxicillin, and among the syrups were azithromycin and ofloxacin, most of them being WATCH antibiotics.

## 3. Discussion

Antibiotic stewardship efforts are hampered by a lack of data on antibiotic availability and usage, particularly from informal markets that are a major source of antibiotics in LMICs. There are several challenges that are involved in collecting medicine data from informal markets [46], including a lack of accurate sampling frames, an absence of sales or patient records and the reluctance of providers to disclose full information about illegal antibiotic sales. We adapted some of the concepts and definitions from the WHO/HAI methodology, and combined these with a cluster sample survey approach [47] in order to first identify IPs, and we then conducted a detailed audit of their antibiotic stocks. We collected data on all of the antibiotic formulations and brands that were available in the clinics (and not just those on the essential drug list), as all have potential significance for AMR. Our study provides one of the first detailed analyses of the diverse range and mix of ACCESS and WATCH antibiotics stocked by IPs, their affordability and the volumes dispensed.

This single cross section has some limitations, as it does not capture seasonality and other temporal variations in antibiotic use, and, being limited to two districts in West Bengal, is not easily generalizable to IPs in other parts of the state and in India. The strength of our study lies in the collection of robust physically observed data on the antibiotics stocked in a cross section of IP clinics, which is a challenging task. A third limitation was that the data on the antibiotics that had been dispensed in the last seven days were based on recall. These could have been subject to recall bias, or they could have been underestimated by the IPs since they are not legally authorized prescribers. However, relative to the total daily patient load reported (median of 25), the scale of antibiotic dispensing is quite plausible. These sales data were collected towards the end of the survey, after our researchers had established good rapport with each IP, in order to minimize bias.

The IPs in our study stocked 42 different types of antibiotics, which included both ACCESS (71%) and WATCH (83%) antibiotics (Supplementary Table S3). There were more WATCH (17) than ACCESS (12) antibiotics stocked, and the remaining 13 were on the WHO’s ‘not recommended’ list. The WATCH antibiotics included third-generation cephalosporins (e.g., cefixime and cefpodoxime), quinolones (e.g., ciprofloxacin and ofloxacin) and macrolides (e.g., azithromycin). The high rate of use of third-generation cephalosporins and quinolones is also reported in another IP study in Madhya Pradesh [40], which is a finding that mirrors an increasing trend in the use of these antibiotic classes in other general outpatient and inpatient settings in India [48]. India has the second highest national consumption of WATCH antibiotics across 76 countries [49]. More than 60% of the antibiotics that are consumed in India are from the WATCH group, compared with the WHO’s target that ACCESS antibiotics should account for 60% of the antibiotics in the overall national consumption. The higher use of some WATCH antibiotics in India has been attributed to their greater availability, their broad-spectrum activity and the convenience of dosing [48]. Our study findings suggest that affordability could also be an important factor. In our study, three of the four affordable tablets (ciprofloxacin, azithromycin and cefixime) and the two affordable syrups/suspensions/drops (azithromycin and ofloxacin) were from the WATCH group (Figure 6).

In India, the central government caps the prices of 851 essential drugs (exclusive of local taxes), including several antibiotics [50]. The median prices of our top five antibiotics were similar to the 2017 government ceiling prices in the Drug Price Control Order [51]. For example, the ceiling price was US$ 5.3 for a 7-day course of amoxicillin potassium clavulanate (US$ 5.5 in our sites), US$ 1.9 for cefixime and amoxicillin (also US$ 1.9 in our sites), US$ 0.8 for azithromycin (US$ 0.9 in our sites) and US$ 0.7 for ciprofloxacin (US$ 0.8 in our sites). In countries where the government does not control the pricing, such as in Malaysia, medicine prices have been found to be much higher [52]. Affordable pricing is necessary in order to ensure access to essential drugs; however, as our small study indicates, this may also lead to the greater consumption of WATCH antibiotics.

In addition, we found low availability of older first-line ACCESS antibiotics, such as co-trimoxazole (trimethoprim/ sulfamethoxazole), ampicillin and doxycycline. Less than 10% IPs stocked these, and none stocked penicillin. These antibiotics have low overall availability in India. One recent study commented that few companies in India manufacture older ACCESS antibiotics [48]. The authors found that only one company manufactured penicillin and benzathine penicillin, whereas 51 manufactured amoxicillin, 112 manufactured amoxicillin–clavulanic acid and 135 manufactured cefixime. However, a few studies from rural community settings [6,7] have reported high levels of resistance against penicillin and ampicillin (but less against gentamycin and amikacin, which are also ACCESS antibiotics), which implies that there may be variations in susceptibility, even amongst older ACCESS antibiotics. This knowledge is needed in order to guide antibiotic choices in different locations.

The presence of the ‘not recommended’ irrational antibiotic combinations that were found in our study also reflected the wider Indian medicine market. Stocked by 21% of the IPs (Figure 2), these are also known as “fixed dose combinations” (FDCs) in India. They are not recommended by the WHO, as their use is not evidence-based, but India has the second highest consumption of these antibiotics in the world (7.5%), after Egypt (9.6%) [49]. They are favoured by senior medical prescribers in India and the pharmaceutical industry [53], and efforts by the Central Drugs Standard Control Organisation to ban FDCs have been challenged by the industry, which has led to long-drawn-out legal battles [54].

We found a large number of brands (278 brands, 74 manufacturers) in our study sites, and several different brands with the same antibiotic ingredients. This too reflects the nature of the medicine market in India, which abounds in branded generics. These are off-patent medicines that companies package and sell under different brands in order to promote brand loyalty [55]. Growth in this market is a major opportunity for Indian firms, with rural areas offering major new revenue segments [56]. We also found substantial variation between the lowest and highest priced brands of each of the top five antibiotics. This interbrand price variation has been found in many formulations, including in antibacterials in India [57]. This is understood to be a consequence of the method that is used to calculate the government’s ceiling price—by averaging the wholesale prices of all of the companies with a market share greater than 1%, and adding a retailer margin of 16%—so that, in effect, the ceiling prices are actually determined by the already existing prices of the popular brands [57]. The brands that were the most stocked by the IPs were priced in-between the lowest and highest priced ones, and they did not have the highest retailer margins. The median percentage markups across the most stocked tablet brands were in the range of 15–24%, which was close to the government’s recommended retailer margin of 16% in the Drugs Price Control Order [50]. In our study’s accompanying in-depth interviews, the IPs explained that the highest priced brands were beyond the payment capacity of most patients, and that the lowest priced ones were perceived to be of doubtful quality [31].

A large percentage of the IPs in our study stocked both tablets (84%) and syrups/suspensions/drops (74%). In general, the syrups/suspensions/drops in our study cost more per adult treatment course than tablets, but, as many of these were paediatric formulations, a smaller (and more affordable) dose would have been required for most users. As paediatric DDDs are not available, we used adult DDDs in our analysis, but the fact that 74% of the IPs stocked syrups/suspensions/drops is indicative of the IPs’ key role in the management of childhood illnesses with antibiotics, as confirmed by other studies [29]. Only 18% of the IPs stocked an injection, which is a positive finding given that the unnecessary use of injections has long been a concern in the informal sector [58].

The IPs in our study were low-volume stockists and dispensers, as was evident from the small numbers of DDDs that they stocked and dispensed. This is also suggestive of suboptimal dispensing, which has implications for fueling AMR. In our affiliated study [31], we describe the challenges that are involved in addressing this and other inappropriate antibiotic practices by IPs in rural India. Regulations that criminalise IPs and strictly enforce the prescription-only sales of antibiotics are difficult to enforce, as the regulators are concerned that these would adversely impact on people’s access to essential healthcare and antibiotics. Moreover, IPs are an important part of India’s pluralistic health system legacy, and an invisible crutch for an expanding private health sector that benefits from training them and receiving referrals from them. IPs are also a critical revenue segment for India’s growing pharmaceutical industry, which aggressively markets and incentivizes IPs’ antibiotic use. Restrictive regulations have not succeeded in curtailing IP practices, but they have been a major barrier to designing practical and effective interventions with IPs. In an ongoing study [59], we have been engaged with multiple stakeholders from the medical, regulatory and industry sectors to codesign an intervention that targets IPs and the drivers of antibiotic use. A key stakeholder recommendation has been to develop optimal antibiotic use guidelines for IPs and other nonphysician health providers, for a limited range of antibiotics and healthcare conditions. If IPs are enabled to use some ACCESS antibiotics in the right way, regulators might find it easier to restrict their use of WATCH antibiotics (and potentially also of RESERVE ones). While national antibiotic use guidelines exist for physicians [60], there are none for IPs, so these need to be developed at the very first step of an intervention. Future research is needed to evaluate the effectiveness of these guidelines for optimizing the IPs’ antibiotic use. Research is also needed to understand the lack of availability of some antibiotics, the ways to make them more available and on strategies to align pharmaceutical manufacturing, marketing and pricing with antibiotic stewardship.

## 4. Materials and Methods

### 4.1. Study Setting

This study was conducted in the state of West Bengal in eastern India. The state is divided into 19 districts; in 13 of these, more than 70% of the population is rural [61]. We purposively selected two districts from these 13: Birbhum (87% rural) from the north, and South24Parganas (74% rural) from the south, which borders the Bay of Bengal [62,63]. South24Parganas has a well-developed urban fringe owing to its proximity to the state’s capital, Kolkata, but it also includes several remote and riverine villages in the mangrove forests of the ‘Sundarbans’ (beautiful forest) [63].

### 4.2. Sampling and Data Collection

Given the lack of an existing sampling frame of IPs, we chose to randomize clusters instead of IPs. Districts in India are divided into administrative blocks, and each block consists of a cluster of villages that is known as a ‘gram-panchayat’. We defined the clusters as gram-panchayats and adopted a census approach [47] by surveying every IP within randomly selected village clusters in each district. There were 310 gram-panchayats, with an estimated 15 IPs per gram-panchayat in South24Parganas, and 167 gram-panchayats with an estimated 7–8 IPs per gram-panchayat in Birbhum. A sample size of 150 IPs per district was calculated, conservatively, assuming a 50% prevalence of the indicator of interest (antibiotic provision) at a 95% confidence interval and a 10% margin of error, with a design effect of 1.5 (to account for variance due to cluster sampling). To obtain this sample size, we randomly selected 11 g-panchayats in South24Parganas and 7 adjacent pairs of gram-panchayats (14 total) in Birbhum. The IPs were defined as providers who did not possess a formal medical degree or diploma, and who provided consultation services and dispensed/prescribed allopathic (biomedical) drugs. Retail pharmacies and government community health workers were excluded. We consulted IP associations and village key informants in order to identify all of the IPs in the study sites.

The survey tool consisted of a provider questionnaire and an antibiotic stock sheet. The provider survey collected information on the IPs’ sociodemographic characteristics, backgrounds, training and the health services provided. The stock sheet collected information on all of the antibiotics stocked by the IPs, including tablets, syrups/suspensions/drops and injections; their brand names and manufacturers’ names; their pack sizes; their retail prices; the IPs’ purchase prices; the amounts dispensed in the last seven days; and the number of patients that were dispensed each antibiotic. The researchers requested that the IPs show all the antibiotics in their stock and transferred information from the packs onto the stock sheet. The purchase price of each antibiotic (price paid by the IP to the wholesaler) and the sales volume, and the patients who received that antibiotic in the last seven days, were based on the IPs’ recall. The principal investigator (PI) trained the data collection team over four days of classroom training, which was followed by a round of field training. The team practiced developing rapport with the providers; the provider survey was completed before filling out the stock sheet because the latter was expected to be more sensitive. The tool was piloted twice in a nonstudy district with 17 IPs, and the final survey was conducted during February–May 2017. Each completed stock sheet was reviewed by the study coordinator, and a random sample was reviewed by the PI.

### 4.3. Analysis

The data were entered into Microsoft Excel and were analysed for the two districts by using Excel and Stata 14. This analysis only included IPs who had antibiotics in stock on the day of the visit and who provided consent (196 out of 291 IPs).

Availability and Sales: The availability was calculated as the percentage of outlets stocking a specific type of antibiotic: by formulation (tablets, syrups/suspensions/drops and injections), by active ingredient and by the ATC classification [41]. The antibiotics were further classified according to the WHO AWaRe categories [10] and by the list of fixed dose combinations that are not recommended by the WHO [10]. We further classified them by the list of drugs that are banned in India [43] and by the Indian drug ‘schedule’, H1. Schedule H1 is a subset of prescription-only medicines that are subjected to additional regulatory controls, such as the maintenance of the sales registers by the pharmacies. The schedule includes several third-generation and last-resort antibiotics, while all of the other antibiotics are in Schedule H [42].

For the five most commonly stocked tablets and the five most commonly stocked syrups/suspensions/drops, we analysed the quantities that were in stock on the day of the visit and that had been dispensed in the last seven days, in terms of the defined daily doses (DDDs). The DDD is the assumed average maintenance dose per day for a drug for its main indication in adults [41]. By the WHO/HAI methodology [37], DDDs can be measured as DDDs/1000 inhabitants, or as median DDDs dispensed/patient. We adopted the latter measure for our study.

Price: For each of the five most stocked antibiotics, the median prices were calculated for a standard treatment dose for an adult for tablets and for syrups/suspensions/drops. For example, for amoxicillin, the assigned DDD is 1500 mg per day, so a standard course of treatment lasting 7 days will include 10,500 mg of amoxicillin. In practice, the duration of antibiotic therapy should depend on the nature of the infection and the response to treatment [44], and it can vary from a single dose for an uncomplicated urinary tract infection, to prolonged treatment for tuberculosis. For the sake of uniformity, we assumed a standard therapy duration of 7 days for amoxicillin/potassium clavulanate, ciprofloxacin and cefixime (the standard duration for adult respiratory infections and other bacterial abdominal/urinary/gastrointestinal infections) [37,44], and of 5 days for azithromycin, as this is the standard duration that is recommended by the WHO [41]. Although some products were specifically targeted at children (e.g., syrups and dry suspensions for paediatric use), for comparability, and since the DDD methodology does not assign paediatric DDDs, we report all the prices on the basis of the DDD for an adult dosage.

The median prices are reported across all brands with a given active ingredient, and for the lowest priced, most stocked and highest priced brands in that category. These data are presented for the five most stocked antibiotics in their tablet and syrup formulations. The prices for injections were calculated per DDD, and not for full courses. Because of the small number of products, these data are presented in the text but not in Table 4.

Percentage markup for the IP: We calculated the percentage markup as the ((retail price–purchase price)/purchase price)) × 100 for the standard treatment dose for an adult for tablets and syrups/suspensions/drops.

Affordability: The WHO/HAI propose assessing the affordability by comparing the median price for a standard adult dose with the daily wage of the lowest paid government worker. In the setting of rural West Bengal, we felt that the minimum daily wage of an unskilled agricultural worker (INR 226/US$ 3.6, from January—June 2017) [45] would be a more appropriate comparator, as our surveyed IPs reported that the majority of their clients were in this category, while government employment was relatively rare. We calculated the number of days’ wages that would be required to cover the median retail price (across all brands) for a standard adult course of treatment for each of the five most common antibiotics in their tablet and syrup/suspension/drop formulations.

## 5. Conclusions

Our study provides new insights into the antibiotics that are stocked by rural IPs in India. The products that were available in the IP clinics reflected many of the characteristics of the larger medicine market in India, including the greater variety of affordable WATCH antibiotics, and several branded generics to choose from. The most stocked brands were priced in between the lowest and highest priced ones and they had more uniform provider markups across the brands, which suggests that markups were not the only consideration in the choice of antibiotics that were stocked; the price-to-user and the perceived product quality were also important factors. Potential interventions to improve the antibiotic practices in this setting will need to address the providers’ economic and social interests in dispensing drugs and antibiotics, and the lack of antibiotic use guidelines for providers at this primary level. Market factors, such as pricing policies for ACCESS and WATCH antibiotics and the restrictions on the number of irrational combinations, will need to be addressed by the government. Antibiotic regulations could be simplified through a closer alignment between the Indian H/H1 schedules and the WHO’s AWaRe classification. National and state governments could consider developing evidence-based policies to increase the availability of older-generation ACCESS antibiotics, and to restrict the use of WATCH antibiotics, corresponding to the local resistance patterns. To enable the responsible use of antibiotics by IPs, health and regulatory authorities will need to enable them to use some ACCESS antibiotics in the right way, while restricting the use of a large variety of broad-spectrum WATCH antibiotics, which is a practical approach that could lead to more effective antibiotic stewardship in LMIC community settings. This needs to be supplemented with market research on the low availability of some ACCESS antibiotics, and the measures to address this gap.

## Figures and Tables

**Table 1:** Informal provider (IP) sociodemographic and practice characteristics.

Characteristics	% of IPs (*n* = 196)
Gender	
Male	98%
Age	
<35 years	23.5%
36–45 years	31.5%
46–55 years	22.5%
>55 years	22.5%
School education	
Up to Class 10	34%
Up to Class 12	31%
Graduate or postgraduate	35%
Years of practice	
<10 years	32%
10.1–20 years	32%
>20 years	36%
Any allied health certification	73%
Worked as a compounder to formal doctors	83%
Operate out of a small clinic	100%
Days per week the clinic is open	
7 days	75%
6 days	21%
5 days or less	4%
Mode of using antibiotics	
Dispense	95%
Prescribe	86%
**Health services**	
Outpatient care for common illnesses (fevers, diarrhea and cold/cough)	97%
Home visits (on call)	92%
Inpatient care	15%
Diabetes	66%
Hypertension	91%
Dental care	91%
Eye care	86%
Wound suturing	90%
Small surgeries (e.g., draining an abscess)	78%
Piles	5%
Delivery care	26%
Abortions	19%
Animal healthcare (mainly cattle and poultry)	40%

**Table 2:** Top 5 antibiotic active ingredients stocked by IPs, by formulation (*n* = 196).

Tablets		Syrups/Suspensions/Drops		Injections	
Antibiotic	% of IPs Stocking	Antibiotic	% of IPs Stocking	Antibiotic	% of IPs Stocking
Amoxicillin	29%	Amoxicillin potassium clavulanate	37%	Amikacin	6%
Amoxicillin potassium clavulanate	23%	Cefixime	30%	Cefotaxime	4%
Ciprofloxacin	20%	Cefpodoxime	13%	Ceftriaxone	4%
Azithromycin	17%	Azithromycin	9%	Ampicillin	1%
Cefixime	15%	Ofloxacin	8%	Gentamicin	1%

**Table 3:** DDDs available, DDDs dispensed in the last seven days * and numbers of patients dispensed * antibiotics for the top 5 antibiotics stocked by IPs, by formulation (*n* = 196).

Antibiotic (Number of IPs Stocking)	DDDs Available, Median (IQR)	DDDs Dispensed Last 7 Days, Median (IQR)	No. of Patients Dispensed Antibiotic Last 7 Days, Median (IQR)	DDDs Dispensed Per Patient, Median (IQR)
**TABLETS**				
Amoxicillin (n = 56)	10 (6, 15)	10 (7, 20)	7 (3, 11)	2 (1, 3)
Amoxicillin potassium clavulanate (n = 45)	10 (6, 12)	7 (5, 20)	4 (2, 7)	2 (2, 3)
Ciprofloxacin (n = 38)	15 (10, 19)	15 (8, 25)	5 (3, 10)	3 (2, 4)
Azithromycin (n = 36)	25 (10, 40)	20 (15, 50)	4 (3, 7)	5 (5, 7)
Cefixime (n = 30)	15 (10, 20)	10 (8, 25)	4 (2, 7)	4 (2, 5)
**SYRUPS/SUSPENSIONS/DROPS**				
Amoxicillin potassium clavulanate (n = 72)	2 (1, 4)	4 (2, 6)	4 (1, 7)	1 (1, 1)
Cefixime (n = 59)	2 (1, 3)	3 (1, 8)	4 (2, 10)	1 (0.50, 1)
Cefpodoxime (n = 26)	1.5 (1.50, 3)	2 (1.5, 7.50)	3 (2, 7)	0.75 (0.75, 0.75)
Azithromycin (n = 18)	4 (2, 6)	6 (2, 10)	3 (1, 5)	2 (2, 2)
Ofloxacin (n = 17)	1.50 (0.75, 3)	2 (1.50, 4.50)	2 (1, 4)	0.75 (0.75, 0.75)
**INJECTIONS**				
Amikacin (n = 13)	1.5 (0.75, 3)	2 (0.50, 4.50)	4 (2, 6)	0.50 (0.25, 0.75)
Cefotaxime (n = 9)	0.19 (0.08, 0. 25)	0.31 (0.13, 0.63)	7 (4, 10)	0.06 (0.01, 0.08)
Ceftriaxone (n = 8)	1.25 (0.70, 1.60)	1.46 (0.22, 5.50)	3 (1, 11)	0.46 (0.19, 0.50)
Ampicillin (n = 2)	2.29 (0.83, 3.75)	2.94 (1.38, 4.50)	17 (5, 30)	0.21 (0.15, 0.30)
Gentamicin (n = 2)	1.91 (0.50, 3.33)	0.75 (0.17, 1.33)	4 (4, 5)	0.18 (0.03, 0.33)

* As reported by providers. Note: Each median value is per the product stocked.

**Table 4:** Median prices of standard courses of treatment for the top 5 antibiotics, across different brands.

Antibiotic (Number of Products Available in Different Clinics)	Brand Name, Manufacturer	Median Retail Price in INR/USD for Standard Course *
**TABLETS**		
**Amoxicillin potassium clavulanate**	**Total brands: 20**	
Lowest priced brand (n = 1)	Medimox Plus, Alkem	89/1.36
Most stocked brand (n = 11)	Clavam 625, Alkem	357/5.49
Highest priced brand (n = 1)	Augmentin 100, Medreich	564/8.67
Price ratio: highest:lowest		6.2
**Cefixime**	**Total brands: 19**	
Lowest priced brand (n = 1)	Sefbactum, Akums	76/1.16
Most stocked brand (n = 6)	Taxim-O, Alkem	125/1.92
Highest priced brand (n = 1)	Noxcef-O, Embark	252/3.87
Price ratio: highest:lowest		3.3
**Amoxicillin**	**Total brands: 11**	
Lowest priced brand (n = 1)	Moxileb, Leben	109/1.67
Most stocked brand (n = 17)	Wymox, Pfizer	125/1.92
Highest priced brand (n = 1)	Moxilium, Biochem	244/3.75
Price ratio: highest:lowest		2.2
**Azithromycin**	**Total brands: 22**	
Lowest priced brand (n = 1)	Zedcin-500, Medmark	21/0.32
Most stocked brand (n = 6)	Azithral, Alembic	66/1.00
Highest priced brand (n = 2)	Trulimax, Pfizer	71/1.09
Price ratio: highest:lowest		3.6
**Ciprofloxacin**	**Total brands: 14**	
Lowest priced brand (n = 1)	CPGLO-500, Arose Pharma	17/ 0.26
Most stocked brand (n = 11)	Cifran, Sun Pharma	48/0.73
Highest priced brand (n = 2)	Cifran, Ranbaxy	108/1.66
Price ratio: highest:lowest		5.6
**SYRUPS/SUSPENSIONS/DROPS**		
**Amoxicillin potassium clavulanate**	**Total Brands: 28**	
Lowest priced brand (n = 1)	Clavactum, Park Pharma	372/5.72
Most stocked brand (n = 25)	Clavam, Alkem	507/7.80
Highest priced brand (n = 1)	Safemox-CL, Magma Allianz	622/9.56
Price ratio: highest:lowest		1.7
**Cefixime**	**Total Brands: 19**	
Lowest priced brand (n = 2)	Zofix, Alembic	203/3.12
Most stocked brand (n = 30)	Taxim-O, Alkem	392/6.03
Highest priced brand (n = 1)	Cefix, Cipla	849/13.06
Price ratio: highest:lowest		4.2
**Cefpodoxime**	**Total Brands: 17**	
Lowest priced brand (n = 1)	Opox-CV 50, Hetero	140/2.15
Most stocked brand (n = 7)	Monocef-O, Aristo	653/10.04
Highest priced brand (n = 1)	Cefpo-CV 50, Finecure	887/13.64
Price ratio: highest:lowest		6.3
**Azithromycin**	**Total Brands: 7**	
Lowest priced brand (n = 1)	Azid, Indi Pharma	65/1.00
Most stocked brand (n = 4)	Azithral, Alembic	99/1.52
Highest priced brand (n = 1)	Zithrocin, Biochem	200/3.07
Price ratio: highest:lowest		3.1
**Ofloxacin**	**Total Brands: 10**	
Lowest priced brand (n = 4)	OFM, Lekar Pharma	117/1.80
Most stocked brand (n = 7)	O2, Medley	420/6.46
Highest priced brand (n = 1)	Powergyl, Cipla	569/8.75
Price ratio: highest:lowest		4.9

* Standard course of treatment was taken as * 7 DDDs for amoxicillin clavulanate, amoxicillin, cefixime and ciprofloxacin, and 5 DDDs for azithromycin [37,41,44]. Injections are not included in this analysis because of the small number of products found.

## Data Availability

The data presented in this study can be made available upon request from the corresponding author. The dataset is not publicly available as it forms part of ongoing research and analysis. It will be made publicly available once the major study publications are completed.

## Supplementary Materials

The following supporting information can be downloaded at https://www.mdpi.com/article/10.3390/antibiotics11040523/s1, Figure S1: Percentage of IPs stocking different types of antibiotics by active ingredient and formulation; Table S1: IPs’ health-related certification, by level of education; Table S2: Antibiotic formulations stocked by IPs with any antibiotic in stock on the day of the visit (*n* = 196); Table S3: Antibiotics stocked by IPs by WHO classifications and Indian regulations.
