# Supplementary material for: Availability, Prices and Affordability of Antibiotics Stocked by Informal Providers in Rural India: A Cross-Sectional Survey
Source: Antibiotics (Basel). 2022 Apr 14;11(4):523. doi: 10.3390/antibiotics11040523 (PMC9026796; doi:10.3390/antibiotics11040523)
Supplement: Supplementary file 1 [file antibiotics-11-00523-s001.zip › antibiotics-1624653-supplementary.pdf]

antibiotics

MDPI

Supplementary Table S1: IPs' health-related certification, by level of education

Education (School and college)	No certification	Pharmacy certification	Laboratory assistant's certification	Certification in basic primary healthcare courses*
Up to Class 10 (n= 66)	38%	11%	1%	50%
Class 11-12 (n= 61)	18%	10%	0%	72%
Graduate/ postgraduate (n=69)	26%	7%	1%	65%
Total (n=196)	28%	9%	1%	62%

*Examples: 'Community Medical Service', 'Rural Medical Practitioner', 'Alternative Medicine', 'Basic Allopathic Medicine', 'Indo-allopathic Medicine'.

Supplementary Table S2: Antibiotic formulations stocked by IPs with any antibiotic in stock on the day of the visit (n=196)

Antibiotics	All forms	Tablets	Syrups/suspensions /drops	Injections
Any antibiotics in stock		165/196 (84%)	146/196 (74%)	36/196 (18%)
Median number of different antibiotics (by active ingredient) stocked (IQR)	3 (2-5)	2 (1-3)	1 (0-2)	0 (0-0)
Median number of different ATC* classes stocked (IQR)	2 (2-3)	2 (1-2)	1 (0-2)	0 (0-0)

* Anatomical Therapeutic Chemical classification system (WHO, 2021)
IQR= Interquartile range

Supplementary Figure S1: Percentage of IPs stocking different types of antibiotics by active ingredient and formulation

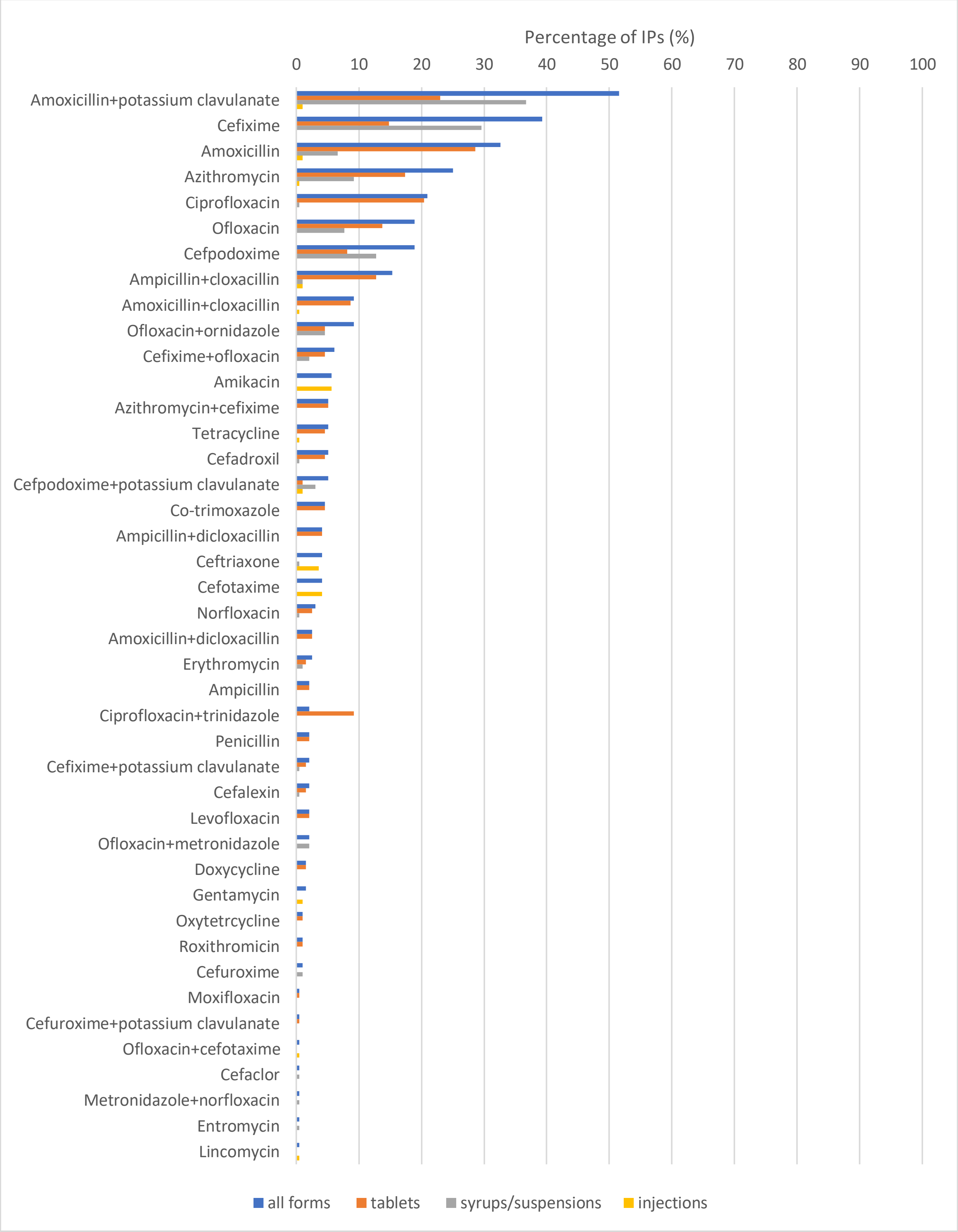

Supplementary Table S3: Antibiotics stocked by IPs by WHO classifications & Indian regulations

Antibiotic	WHO classifications			Indian Drugs & Cosmetics Act	
	ACCESS	WATCH	Not recommended	Schedule H1*	Banned
Amoxicillin potassium clavulanate	■				
Cefixime		■		■	
Amoxicillin	■				
Azithromycin		■			
Ciprofloxacin		■			
Ofloxacin		■			
Cefpodoxime		■			
Ampicillin + cloxacillin			■		
Amoxicillin + cloxacillin			■		■
Ofloxacin + ornidazole			■		
Cefixime + ofloxacin			■		
Amikacin	■				
Azithromycin + cefixime			■		■
Tetracycline	■				
Cefadroxil	■				
Cefpodoxime + potassium clavulanate			■	■	
Co-trimoxazole	■				
Ampicillin + dicloxacillin			■		
Ceftriaxone		■		■	
Cefotaxime		■		■	
Norfloxacin		■			
Amoxicillin + dicloxacillin			■		
Erythromycin		■			
Ampicillin	■				
Ciprofloxacin + tinidazole			■		
Penicillin	■				
Cefixime + clavulanic acid			■		
Cefalexin	■				
Levofloxacin		■		■	
Ofloxacin + metronidazole			■		
Doxycycline	■				
Gentamycin	■				
Oxytetracycline		■			
Roxithromycin		■			
Cefuroxime		■			
Moxifloxacin		■		■	
Cefuroxime potassium clavulanate			■		
Ofloxacin + cefotaxime**		■			
Cefaclor		■			
Metronidazole + norfloxacin			■		■
Enteromycin (chloramphenicol)	■				
Lincomycin		■			

* This Schedule contains a restricted set of 46 prescription drugs including third and fourth generation cephalosporins, carbapenems, newer fluoroquinolones and first- and second-line antitubercular drugs. Sales of Schedule H1 drugs are more strictly monitored than of Schedule H drugs (GOI, 2016a).

**A combination of two WATCH antibiotics but not listed in the 'Not Recommended' or 'Banned' products.
